# The *Arabidopsis* Golgi-localized GDP-L-fucose transporter is required for plant development

**DOI:** 10.1038/ncomms12119

**Published:** 2016-07-06

**Authors:** Carsten Rautengarten, Berit Ebert, Lifeng Liu, Solomon Stonebloom, Andreia M. Smith-Moritz, Markus Pauly, Ariel Orellana, Henrik Vibe Scheller, Joshua L. Heazlewood

**Affiliations:** 1 Joint BioEnergy Institute, Physical Biosciences Division, Lawrence Berkeley National Laboratory, Berkeley, California 94702, USA; 2 ARC Centre of Excellence in Plant Cell Walls, School of BioSciences, The University of Melbourne, Melbourne, VIC 3010, Australia; 3 Department of Plant and Environmental Sciences, Faculty of Science, University of Copenhagen, Copenhagen DK-1871, Denmark; 4 Department of Plant and Microbial Biology, University of California, Berkeley, California 94720, USA; 5 Centro de Biotecnología Vegetal, Facultad de Ciencias Biológicas, Universidad Andrés Bello, Santiago RM 8370146, Chile; 6 Fondo de Areas Prioritarias Center for Genome Regulation, Santiago RM 8370146, Chile

## Abstract

Nucleotide sugar transport across Golgi membranes is essential for the luminal biosynthesis of glycan structures. Here we identify GDP-fucose transporter 1 (GFT1), an *Arabidopsis* nucleotide sugar transporter that translocates GDP-L-fucose into the Golgi lumen. Using proteo-liposome-based transport assays, we show that GFT preferentially transports GDP-L-fucose over other nucleotide sugars *in vitro*, while *GFT1*-silenced plants are almost devoid of L-fucose in cell wall-derived xyloglucan and rhamnogalacturonan II. Furthermore, these lines display reduced L-fucose content in *N*-glycan structures accompanied by severe developmental growth defects. We conclude that GFT1 is the major nucleotide sugar transporter for import of GDP-L-fucose into the Golgi and is required for proper plant growth and development.

## Abstract (web summary)

Nucleotide sugars are transported from the cytoplasm to the Golgi lumen where they are incorporated into cell wall polysaccharides and used for glycosylation of proteins and lipids. Here the authors identify GFT1, an *Arabidopsis* Golgi-localized GDP-fucose transporter that is required for plant growth and development

## Graphical abstract

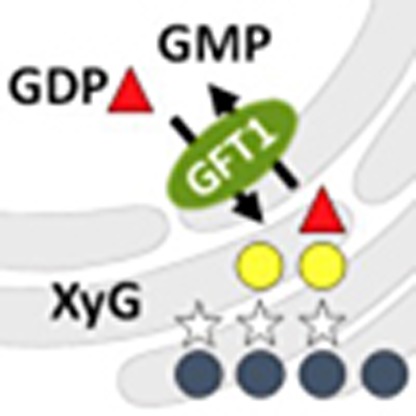

The initial stages of the plant secretory system include the endoplasmic reticulum (ER) and the Golgi apparatus. These compartments are essential for the biosynthesis of non-cellulosic cell wall polysaccharides, and the glycosylation of proteins and lipids. These processes are mediated by glycosyltransferases that require activated substrates such as nucleotide sugars. While most nucleotide sugars are linked to uridine-5′-diphosphate (UDP), some important nucleotide sugars are linked to guanosine-5′-diphosphate (GDP). Plants contain a number of GDP sugars including GDP-D-mannose (GDP-Man), GDP-L-fucose (GDP-Fuc), GDP-D-glucose (GDP-Glc) and GDP-L-galactose (GDP-Gal)^1^. GDP-Man is the substrate for the mannosylation of glycosylinositolphosphoceramides (GIPCs)^2^, dolichol-P^3^, *N*-linked glycans^4^ and together with GDP-Glc, important for the biosynthesis of the cell wall polymer glucomannan^5^. GDP-Fuc is essential for the fucosylation of xyloglucan^6^, *N*-linked glycans^7^ and arabinogalactan proteins^8^, while both GDP-Fuc and GDP-Gal are substrates for the biosynthesis of the pectic polymer rhamnogalacturonan II (RG-II)^9^.

In contrast to most nucleotide sugars, which are generated by sequential interconversions from UDP-Glc, GDP-Fuc is derived from GDP-Man. GDP-Fuc formation requires the combined activities of the GDP-D-mannose-4,6-dehydratase (GMD) and the GDP-4-keto-6-deoxy-D-mannose-3,5-epimerase-4-reductase^10^. A mutation in the *MURUS 1* (*MUR1*) gene, which encodes an isoform of GMD^11^, results in plants with cell walls almost devoid of α-L-fucosyl residues that are partially replaced by α-L-galactosyl residues in xyloglucan^12^,^13^ and glycoproteins^14^. The *mur1* mutation also affects the cell wall polymer RG-II, resulting in truncations to sidechain A^9^ and sometimes L-fucosyl residues substituted with L-galactosyl residues^15^. The *mur1* mutants exhibit a severe dwarf phenotype with rosette leaves more brittle compared with wild-type plants. A second *Arabidopsis* gene also involved in the fucosylation of a cell wall polymer was later identified and named *MURUS2* (*MUR2*)^6^. Mutants have a reduction in total cell wall L-fucose content, which is ∼50% of that found in wild-type plants. MUR2 is involved in fucosylation of xyloglucan and the L-fucose content in that particular polymer is reduced to ∼1% in the *mur2* background compared to wild-type plants. *MUR2* encodes a Golgi-localized fucosyltransferase later named FUCOSYLTRANSFERASE 1 (FUT1) (ref. 6). In contrast to *mur1* mutants, no defects in growth or cell wall strength were observed in *mur2* plants. Similarly, *fut4* and *fut6* mutants that are defective in arabinogalactan fucosylation do not exhibit phenotypic differences under standard growth conditions when compared with wild-type plants^8^,^16^.

All GDP sugars are biosynthesized in the cytosol and require transport into the Golgi or ER lumen to be made available for glycosylation reactions. To date, a number of nucleotide sugar transporters (NSTs) have been characterized in plants^17^,^18^,^19^,^20^,^21^,^22^. However, the only GDP sugar transporter unambiguously characterized is the GDP-Man transporter GOLGI-LOCALIZED NUCLEOTIDE SUGAR TRANSPORTER 1 (GONST1) from *Arabidopsis*. GONST1 was initially identified based on its homology to GDP sugar transporters identified in other organisms^23^. Several studies have established that GONST1 localizes to the Golgi apparatus, and that it can functionally complement the yeast vanadate resistance glycosylation (*vrg4-2*) GDP-Man transporter mutant^23^,^24^. More recently, it has been shown that GONST1 can transport multiple GDP sugars *in vitro*, and that *gonst1* mutants are dwarfed and can develop spontaneous leaf lesions; however, their cell walls are undistinguishable from wild-type plants^2^. A more detailed characterization of the *gonst1* mutant revealed that GIPC head groups are altered in the mutant, indicating that GONST1 function is essential for the mannosylation of GIPCs *in vivo*^2^. The loss-of-function mutant analyses also suggests that GONST1 is a specific GDP-Man transporter, which is most likely not responsible for the transport of GDP-Fuc and other GDP sugars *in planta*.

The phenotypes obtained for the *mur1* mutants indicate that the availability of GDP-Fuc is critical for normal plant development. Since GDP-Fuc is synthesized in the cytosol but is utilized in the Golgi lumen, a transporter is required to cross the Golgi membrane. To date, very little is known regarding the transport of this GDP sugar in plants other than a requirement for GDP-Fuc transport for the fucosylation of xyloglucan^25^. This study identifies and biochemically characterizes an *Arabidopsis* Golgi-localized GDP-Fuc transporter (AT5G19980), previously named GONST4 according to its homology to other GDP sugar transporters. We show that GONST4 specifically transports GDP-Fuc and not GDP-Man, and our *in vivo* analysis supports its role as the primary Golgi GDP-Fuc transporter. Due to this newly attributed function, we have revised its name to GDP-fucose transporter 1 (GFT1).

## Results

### GFT1 and the *Arabidopsis* NST family

**Figure 1:**
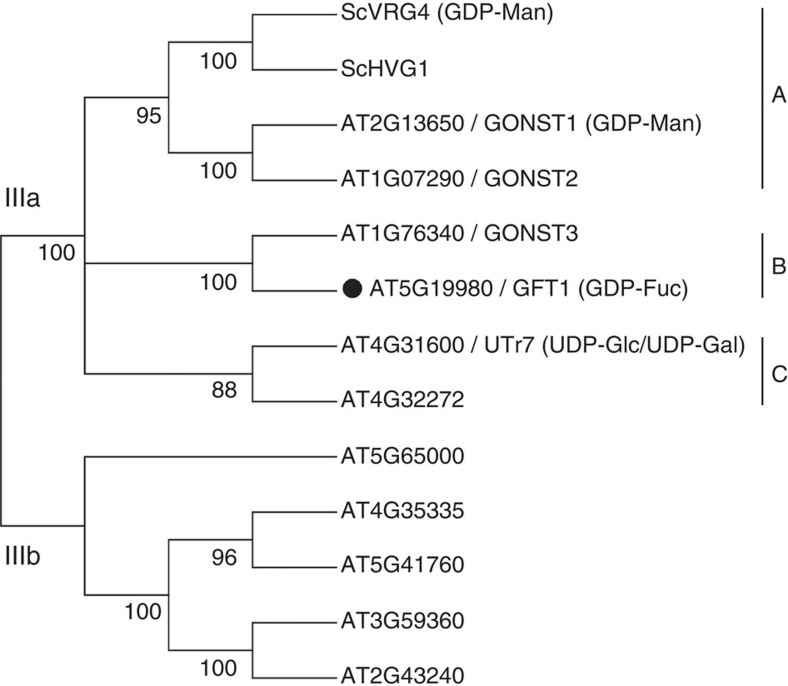
Phylogenetic tree of the *Arabidopsis* NST sub-family III. Full-length amino-acid sequences were aligned and the phylogenetic tree generated using MEGA6. Clades were assigned as previously reported^20^. The yeast GDP-Man transporter ScVRG4 and its paralogue ScHVG1 have also been included in the phylogenetic analysis. The sub-clade IIIa contains the previously characterized *Arabidopsis* GDP-Man transporter GONST1 (ref. 23) and UDP-Glc/UDP-Gal transporter UTr7 (ref. 27). Only bootstrap values >50% are shown.

The previously identified GONST sub-family is part of clade III of the NST/TPT family^20^. A phylogenetic analysis of the full-length protein sequences of the *Arabidopsis* NST clade III members and the previously characterized *Saccharomyces cerevisiae* (yeast) GDP-Man transporter VRG4 (ref. 26) and its paralogue, which is termed homologous to VRG4 (HVG1), indicate that both GONST1 (AT2G13650) and GONST2 (AT1G07290) cluster together with VRG4 and HVG1 to form a sub-clade (A), while GONST3 (AT1G76340) and GFT1 (AT5G19980) form a sub-clade (B). A further sub-clade (C) contains two additional candidates, one of which UTr7 (AT4G31600) has been identified as a UDP-Glc/UDP-Gal transporter^27^ (Fig. 1). GFT1 shares 49% identity with GONST3, and 22 and 21% identity to GONST1 and GONST2, respectively. In comparison, GONST1 and GONST2 share 64% identity at the amino-acid level, while across species, GONST1 shares 31% identity with VRG4 and 32% identity with HVG1. Members of sub-clade (C) share 9–21% identity in their amino-acid sequences when compared with all other members of clade III of the *Arabidopsis* NST family.

### *GFT1* is ubiquitously expressed and localizes to the Golgi

**Figure 2:**
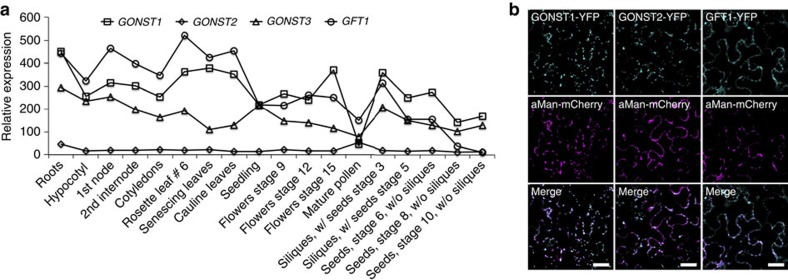
Expression pattern and sub-cellular localizations of *Arabidopsis* GONST members. (**a**) Expression patterns of *GFT1* and *GONST1-3* in different *Arabidopsis* organs and developmental stages using the AtGenExpress developmental data set^28^. (**b**) Sub-cellular localizations of GFT1, GONST1 and GONST2. C-terminal YFP fusion constructs were transiently co-expressed with the aMan-mCherry Golgi marker in *N. benthamiana* leaves. Scale bar, 25 μm.

*In silico* expression analysis of publicly available microarray data comprising the AtGenExpress developmental data set^28^ revealed relatively high and ubiquitous expression of *GFT1*. Similarly, *GONST1* and *GONST3* are ubiquitously expressed at slightly lower levels compared with *GFT1*. In contrast, the *GONST2* transcript appears to be expressed at very low levels with slightly elevated expression in mature pollen (Fig. 2a). These expression levels are also reflected in the total expressed sequence tag (EST) counts for the GONST clade members. *GFT1*, *GONST1* and *GONST3* are all represented by >20 ESTs, while there is only a single EST allocated to the *GONST2* locus. To establish and confirm the sub-cellular localizations of GFT1 and remaining GONST clade members, we generated C-terminal yellow fluorescent protein (YFP) fusions and expressed them transiently in *Nicotiana benthamiana* leaves. The GFT1, GONST1 and GONST2 fusion proteins localized to Golgi-like punctate structures and co-localized with the Golgi marker α-mannosidase I (aMan; Fig. 2b). These results confirm previous Golgi localizations for GONST1 (ref. 23) and GFT1 (refs 24, 29). Despite several attempts, we were unable to express and localize GONST3-YFP in *N. benthamiana* leaves using this transient localization assay.

### Establishing the *in vitro* function for GFT1

**Figure 3:**
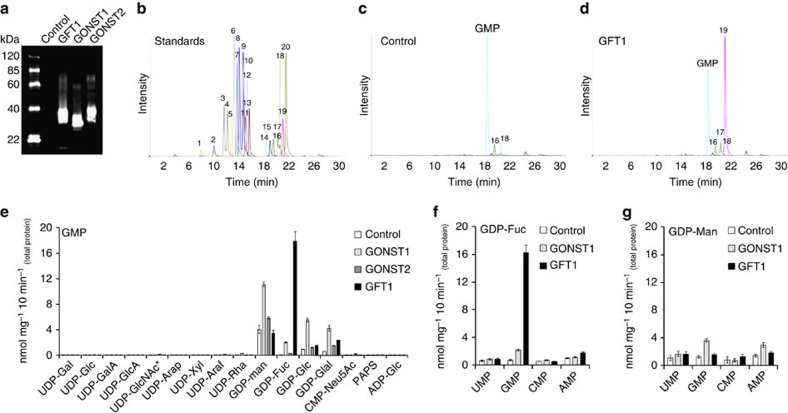
LC-MS/MS analysis of nucleotide sugar transport activities for GFT1 and other GONST members. (**a**) Immunoblot analysis of empty vector, GFT1, GONST1 and GONST2 expression in proteo-liposomes (2.5 μg protein per lane). (**b**) Separation of a 20 nucleotide/nucleotide sugar mix (1, CMP; 2, UMP; 3, UDP-GalA; 4, UDP-GlcA; 5, CMP-Sia; 6, UDP-Ara*p*; 7, UDP-Rha; 8, UDP-Gal; 9, UDP-Glc; 10, UDP-Xyl; 11, UDP-GlcNAc/GalNAc; 12, UDP-Ara*f*; 13, PAPS; 14, GMP; 15, AMP; 16, GDP-Man; 17, GDP-Gal; 18, GDP-Glc, 19, GDP-Fuc; 20, ADP-Glc). LC-MS/MS analysis of proteo-liposomes derived from (**c**) yeast transformed with the empty vector (control) or (**d**) yeast expressing GFT1 after simultaneous incubation with 16 nucleotide sugar substrates. (**e**) Quantification of nucleotide sugar transport into proteo-liposomes pre-loaded with 10 mM GMP. (**f** and **g**) Exchange substrate specificities of GFT1 and GONST1. Proteo-liposomes were loaded with 1 mM GDP-Fuc (**f**) or 1 mM GDP-Man (**g**) and incubated with a mix of UMP, GMP, CMP, AMP at a concentration of 200 μM. Data are mean and s.d. of *n*=4 assays.

To assess GFT1 function, we heterologously expressed the protein in yeast and determined its capacity to transport nucleotide sugars using a recently established approach^20^,^21^. Previous work had indicated that GFT1 had the capacity to partially complement the yeast GDP-Man transporter mutant *vrg4* (ref 24). As a consequence, we also assessed the ability of the previously characterized GDP-Man transporter, GONST1 and its paralogue GONST2 to transport nucleotide sugars using the *in vitro* transporter assay. Unfortunately, as previously outlined for the transient localization assay, we were also unable to express the GONST3 protein in yeast. The corresponding genes were expressed in yeast and microsomal proteins were reconstituted into liposomes for transporter assays. Immunoblot analysis confirmed the presence of each protein (Fig. 3a). Subsequently, proteo-liposomes pre-loaded with either uridine-5′-monophosphate (UMP), guanosine-5′-monophosphate (GMP), cytidine-5′-monophosphate (CMP) or adenosine-5′-monophosphate (AMP) were incubated with a mixture of 16 nucleotide/nucleotide sugar substrates (Fig. 3b). Non-incorporated substrates were removed by gel filtration, and the content of the liposomes after *in vitro* transport was analysed by liquid chromatography–tandem mass spectrometry (LC-MS/MS; Fig. 3c, d). Our results confirmed the previous finding that GONST1 functions preferentially as a GDP-Man transporter^23^ and perhaps as a general GDP sugar transporter with the capacity to transport comparably lower amounts of GDP-Fuc, GDP-Glc and GDP-Gal (Fig. 3e), as has been recently reported^2^. In contrast, GONST2 was limited in its capacity to transport any NDP sugar substrates using the assay (Fig. 3e). Although transport of GDP sugars could be observed in the GONST2 assay, this is likely the result of endogenous transporter activity present in the yeast microsomal preparations, since the incorporation levels were similar to those observed in control reactions (yeast transformed with the empty vector). Our analysis of GFT1 indicated that this NST exhibited the highest activity with GDP-Fuc in comparison with the control, GONST1 and GONST2. The data also show that GFT1 preferentially transported GDP-Fuc in our *in vitro* assay, since no side activities with GDP-Man or GDP-Glc were observed. A minor activity was observed with GDP-Gal, but this was quite low in comparison with the transport of GDP-Fuc (Fig. 3e). All transport was strictly dependent on the presence of GMP as exchange substrate, since no significant transport was observed when proteo-liposomes were pre-loaded with UMP, CMP or AMP (Supplementary Fig. 1). Furthermore, only GMP was accepted as exchange substrate when proteo-liposomes were pre-loaded with GDP-Fuc and then incubated with a mix of the four NMPs (Fig. 3f). No significant transport of any NMP was observed in GFT1-containing proteo-liposomes pre-loaded with GDP-Man (Fig. 3g).

### Kinetic parameters of GFT1 and GONST1

**Figure 4:**
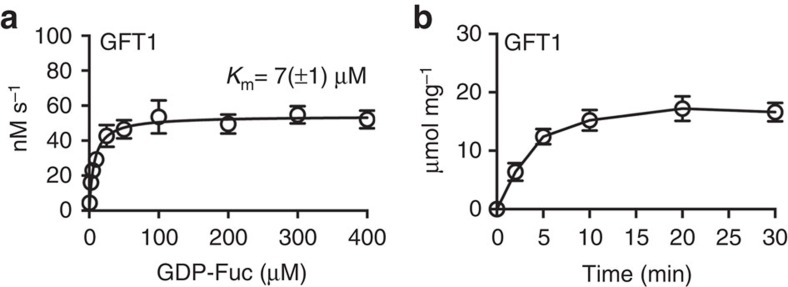
Concentration- and time-dependent transport activities for GFT1. (**a**) Proteo-liposomes, pre-loaded with 10 mM GMP, were incubated with GDP-Fuc at varying concentration (0.5–400 μM) for 2 min at 25 °C. (**b**) Proteo-liposomes, pre-loaded with 10 mM GMP, were incubated with GDP-Fuc at a concentration of 50 μM for the indicated time points at 25 °C. Values are normalized to the actual NST content present in proteo-liposome preparations (Supplementary Table 1). Data are the mean and s.e.m. of *n*=4 assays.

To support an *in vivo* role for GFT1 as the *Arabidopsis* GDP-Fuc transporter, we undertook a detailed examination of the kinetics of GDP-Fuc transport. The GFT1-mediated transport of GDP-Fuc was saturable in a concentration- and time-dependent manner (Fig. 4a,b). The determination of kinetic parameters for GFT1 revealed an apparent *K*_m_ for GDP-Fuc of 7 μM with a turnover rate of ∼4 s^−1^ (Table 1). The apparent *K*_m_ of GONST1 for GDP-Fuc was 76 and 17 μM for GDP-Man (Table 1). This reported *K*_m_ for GDP-Man is consistent with a previous report that estimated *K*_m_ of 26 μM for GONST1 for GDP-Man^30^. Previously, we determined the GDP-Fuc content in various *Arabidopsis* organs^20^. GDP-Fuc concentrations are in the range of 5–20 pmol mg^−1^ dry weight, while the GDP-Man concentrations range from 10 to 50 pmol mg^−1^ dry weights. Assuming the plant vacuole comprises the majority of the aqueous content of a plant cell, the dry weight would be equivalent to the volume of the cellular contents (μl). Accordingly, these cellular GDP-Fuc and GDP-Man concentrations can be considered to be approximately in the μM range. Thus, we estimate that the *K*_m_ value for GDP-Fuc of GFT1 (7 μM) determined using the *in vitro* assay is within physiological range. Similarly, the *K*_m_ value for GDP-Man of GONST1 (17 μM) would also be consistent with physiological concentrations. However, the *K*_m_ value for GDP-Fuc transport of GONST1 (76 μM) is significantly higher than the physiological concentration of GDP-Fuc, indicating that GONST1 does not likely play a significant role as GDP-Fuc transporter *in vivo*.

### The role of GFT1 *in planta*

**Figure 5:**
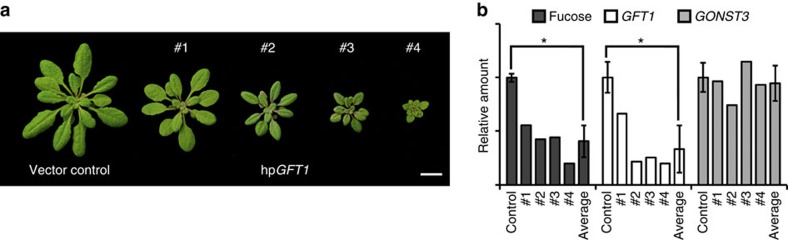
Morphological phenotypes and biochemical characterization of hp*GFT1* lines. (**a**) Representative phenotypes (cohorts) of plants transformed with a hairpin construct specifically targeting *GFT1* expression compared with control plants (plants transformed with the empty vector). Resultant plants were assigned to four categories based on the severity of the phenotype. Scale bar, 10 mm (**b**) Total L-fucose content from cell wall extracts (Fucose) derived from hp*GFT1* lines and the corresponding reductions in *GFT1* expression in these lines. *GONST3* expression was not significantly affected in the hp*GFT1* lines. The control values are mean and s.d. of three independent measurements (*n*=3). The values for the hp*GFT1* lines are the mean of each cohort analysed in triplicate. The ‘average' is the mean and s.d. (*n*=4) of the data from the four independent hp*GFT1* cohorts. Fucose content (*P*<0.003) and *GFT1* transcript levels (*P*<0.005) are significantly different by Student's *t*-test. Values are expressed relative to control lines (plants transformed with the empty vector).

To evaluate the *in vivo* function of GFT1, two putative T-DNA insertion lines were obtained with predicted insertions in the single exon at locus AT5G19980. However, we were unable to identify a T-DNA insertion within the corresponding SAIL_400_E04 and SALK_094857 populations. As a consequence, we used an RNA interference approach through the construction of a hairpin construct specifically targeting *GFT1* transcripts. A total of 75 independent hairpin *GFT1* lines (hp*GFT1*) were selected, and classified into four categories (cohorts) each of which were characterized by varying growth phenotypes (Fig. 5a). Remarkably, ∼50% of the plants died after selection. These plants displayed the strongest morphological defects (10 days post germination) and exhibited severe growth inhibition before death. Analysis of *GFT1* transcript levels from lines in these four categories using quantitative PCR revealed significant reductions in *GFT1* expression correlating with the observed severe developmental defects compared with the control plants transformed with the empty vector (Fig. 5). In comparison, transcript levels of *GONST3*, the closest *GFT1* homologue, were not significantly affected in these plant lines indicating that the hp*GFT1* construct specifically targets *GFT1* expression (Fig. 5b). The reductions in the amounts of *GFT1* transcripts and presumably GDP-Fuc transport did not affect the nucleotide sugar levels, specifically the levels of GDP-Fuc, in hp*GFT1* plants (Supplementary Table 2).

**Figure 6:**
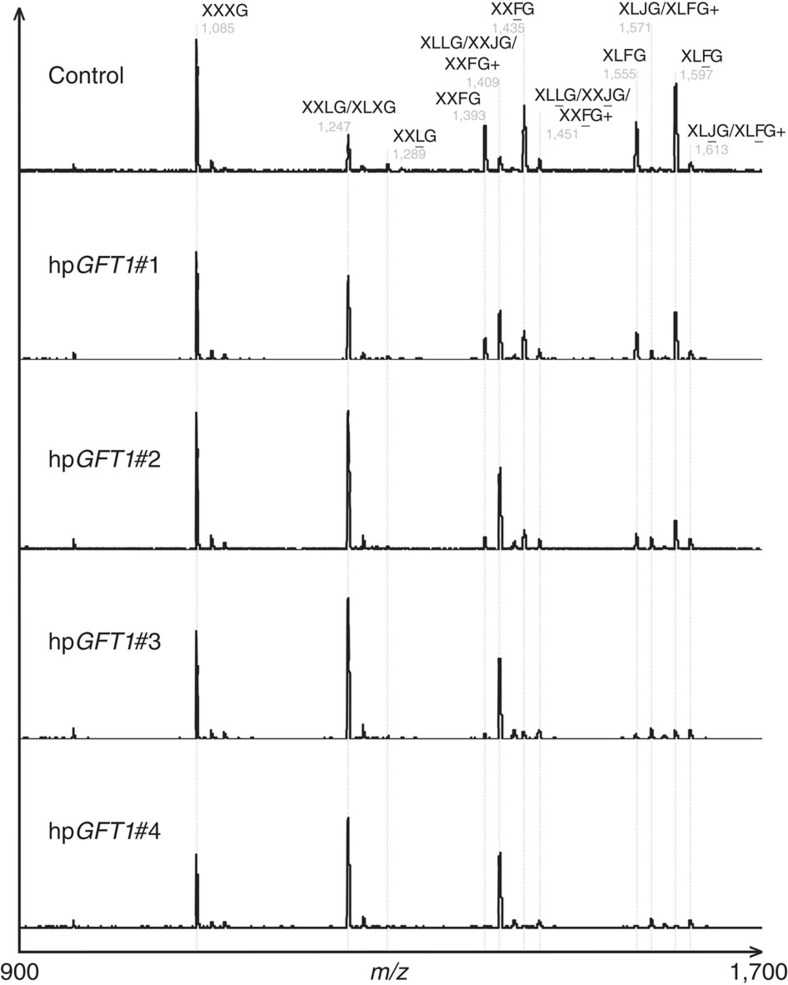
Xyloglucan structures in hp*GFT1* lines. Abundances of xyloglucan oligosaccharides in cell wall material derived from hp*GFT1* lines were determined by oligosaccharide mass profiling (OLIMP). One-letter code nomenclature of oligosaccharides according to ref. 32. All masses are [M+Na^+^] except those labelled (+) which indicates [M+K^+^]. The analysis was carried out in triplicate (Supplementary Table 4).

Analysis of the monosaccharide composition of cell wall extracts revealed considerable reduction in total cell wall-derived L-fucose content (up to 80%) that correlated with the observed reduction in *GFT1* expression (Fig. 5b). To investigate which cell wall polymers are most affected by the downregulation of *GFT1* expression, we determined the monosaccharide composition of sequentially extracted cell wall material derived from hp*GFT1* lines (Supplementary Table 3). The analysis revealed a significant reduction (∼70%) in the L-fucose content in the pectin-enriched CDTA and Na_2_CO_3_ fractions. The reduction in cell wall L-fucose levels was further detected in the xyloglucan-enriched fractions (1 and 4 N KOH). Moreover, in the 4 N KOH fractions of the most severe line hp*GFT1*#4, no residual L-fucose could be detected (Supplementary Table 3). While a significant increase in the proportion of galactose (Gal) in the 4 N KOH fractions was observed in the hp*GFT1* lines, no other sugar varied. Results from the compositional analysis of fractionated cell wall material were further confirmed by oligosaccharide mass profiling (OLIMP). To understand how the reduction in L-fucose content specifically affects the composition of xyloglucan, we enzymatically digested cell wall material from the hp*GFT1* lines with a xyloglucanase and analysed the solubilized oligosaccharide mixtures using matrix-assisted laser desorption/ionization time-of-flight mass spectrometry. The results show that the xyloglucan profiles from hp*GFT1* lines clearly differ from those of control plants transformed with the empty vector. The relative abundance of fucosylated oligosaccharides such as XXFG and XLFG as well as the corresponding acetylated versions XXFG and XLFG^31^ released from cell wall material of the hp*GFT1* lines was significantly decreased compared with the control. Notably, in the lines with the strongest reduction of *GFT1* expression (hp*GFT1*#4), no fucosylated oligosaccharides were detectable (Fig. 6; Supplementary Table 4). Concurrent with the reduced abundance of fucosylated oligosaccharides, non-fucosylated but galactosylated oligosaccharides (XXLG/XLXG and XLLG/XXJG) increased in the lines with reduced *GFT1* expression. The OLIMP analysis was unable to determine whether L-galactose was present in xyloglucan in the hp*GFT1* lines due to the similarities in oligosaccharide masses. The resultant L-galactose containing oligosaccharide (XXJG) has previously been shown to occur in *mur1* mutants^13^.

**Figure 7:**
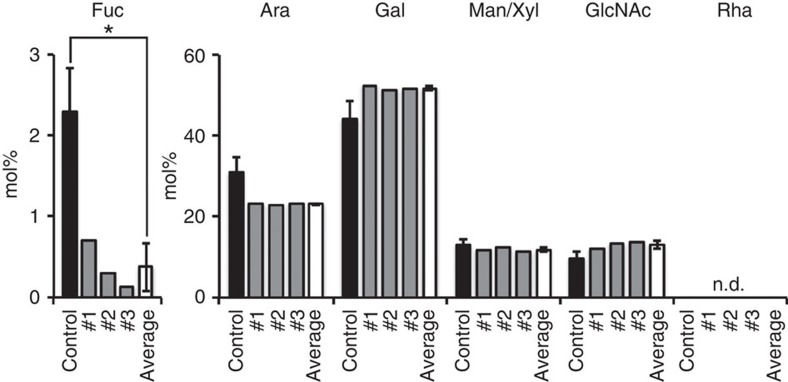
Protein fucosylation in hp*GFT1* lines. Monosaccharide content (mol %) of trifluoroacetic acid-hydrolysed total protein extracted from hp*GFT1* lines measured by anion exchange chromatography. The control values are mean and s.d. of four independent analyses (*n*=4). The values for the hp*GFT1* lines represent the mean of each cohort analysed in triplicate. The ‘average' is the mean and s.d. (*n*=3) of data from the three independent hp*GFT1* cohorts. Fuc levels are significantly different (*P*<0.0004) by Student's *t*-test, as were the sugars Ara (*P*<0.009), Gal (*P*<0.02), Man/Xyl (*P*<0.03) and GlcNAc (*P*<0.005) to a lesser degree.

To examine whether GFT1 plays a role in the fucosylation of proteins, we analysed the monosaccharide content of total protein extracted from hp*GFT1* lines from phenotypic categories 1, 2 and 3 (Fig. 7). A clear reduction in L-fucose was observed in the protein extracts indicating reduced protein fucosylation. Cell wall contamination from pectic material was minimal based on the absence of rhamnose in these samples. Last, to specifically determine the impact on fucosylation of *N*-linked glycans, we analysed total protein with antibodies raised against plant *N*-glycan xylosyl and fucosyl epitopes (Supplementary Fig. 2). As anticipated, xylosylation levels of glycoproteins in the hp*GFT1* lines were not correlated with the severity of the phenotypes. Overall, when compared with control plants transformed with the empty vector, we observed reductions in the fucosylation signal from proteins derived from hp*GFT1* lines from the three categories concomitant with the severity of the observed phenotype (Fig. 5a). The results indicate that the reduction in *GFT1* expression also had a clear impact on the levels of protein fucosylation in plants.

## Discussion

A family of NST-related proteins from *Arabidopsis* termed GONST2, 3, 4 (GFT1) and 5 had been previously been assigned as putative GDP-Man transporters based on the sequence identity to the GDP-Man transporter GONST1 (ref. 24). Previous work had identified GONST1 and GONST2 as GDP-Man transporters and speculated that GFT1 (GONST4) could transport GDP-Man. However, since it could not completely rescue the GDP-Man transport defective yeast mutant *vrg4-2*, it was concluded that it may transport other nucleotide sugars *in planta*^24^. In this study, we have shown that GFT1 (GONST4) is a Golgi-localized transporter with preferential transport activity for GDP-Fuc in plants. We also present evidence that GFT1 function is indispensable for plant growth and development, since plants with significantly reduced GFT1 expression are severely dwarfed and do not grow normally.

The sub-cellular localization of GFT1, GONST1 and GONST2 indicated that all three proteins are located in the Golgi apparatus. This localization is consistent with their function as Golgi NSTs, since the synthesis of D-mannose and L-fucose-containing glycans occurs in this compartment^6^,^32^. Our localization data also confirm previous findings for GFT1 and GONST1 (ref. 24); however, this represents the first localization evidence for GONST2. Given the assigned functional role for GFT1 in transporting GDP-Fuc for lumenal fucosylation reactions, we wanted to confirm its Golgi location and exclude compartments, such as the ER, that could potentially also support this function. Consequently, we re-assessed the sub-cellular localization of GFT1 in *N. benthamiana* using both ER and Golgi markers (Supplementary Fig. 3). We observed a consistent overlap of GFT1-YFP with the Golgi marker with scatter plots indicating a positive correlation. This was further supported by a thresholded Manders' coefficient (tM1 and tM2) analysis, which indicated a high level of overlap between fluorescent signals (Supplementary Fig. 3c). In contrast, we observed a poor correlation between GFT1-YFP and the ER marker (Supplementary Fig. 3b), which was further confirmed after a thresholded Manders' coefficient analysis (Supplementary Fig. 3c).

The transcripts for *GONST1*, *GONST3* and *GFT1* are found ubiquitously throughout plant development (Fig. 2). In contrast, *GONST2* expression levels appear to be very low, which is also supported by the scarce EST evidence, suggesting that GONST2 may not have a major role in most plant organs with the exception of those where some expression is observed, such as mature pollen. Indeed, while GONST2 appeared to be capable of partially complementing the yeast GDP-Man transporter mutant *vrg4-2* (ref. 24), the *in vitro* assay demonstrated negligible GDP-Man transport and our results show that it is incapable of *in vitro* transport of GDP-Fuc. While *GONST2* exhibits minimal expression, *GONST3* is expressed throughout the plant and is homologous to GFT1 with a 49% identity. The gene model, which contains no intron, is supported by multiple ESTs and a complementary (cDNA), but despite repeated attempts, we were unable to clone *GONST3* from cDNA according to this gene model. Similar difficulties have been reported previously^24^, which is consistent with our results and suggests that the annotated gene model for *GONST3* may be incorrect. Interestingly, a recent report biochemically characterized the GONST3 orthologue from grapevine (VvGONST-A) as a GDP-Man transporter^33^, perhaps providing some functional evidence for this locus in *Arabidopsis*. On the other hand, the same study also reports that a grapevine orthologue of GFT1 (VvGONST-B), is a GDP-Glc transporter and exhibits no GDP-Fuc transport^33^. Since the two proteins share high levels of sequence identity (71%), it would be interesting to assess VvGONST-B using our transport assay. It appears that GFT1-like proteins are ubiquitously present in plants; however, mammalian genomes do not appear to contain orthologues of GFT1. Instead, the protein SLC35C1 has been shown to be a GDP-Fuc transporter in mammalian cells^34^ and this protein does not have any close homologues in plants. Thus, the specificity for GDP-Fuc transport has apparently evolved convergently in animals and plants.

To support the *in vitro* function of GFT1, we attempted to identify loss-of-function mutants. However, we were unable to confirm a corresponding T-DNA insert in either of the available lines, both of which had predicted inserts in the single exon of the *GFT1* locus. To overcome this lack of an appropriate knockout line, we generated an RNA interference construct specifically targeting *GFT1* transcripts. Analysis of randomly selected plant lines by quantitative PCR from four phenotypic categories revealed varying degrees of suppression of *GFT1* expression. Consistent with the specific *in vitro* GDP-Fuc transport activity, the reduction in *GFT1* expression strongly correlated with a reduction in L-fucose in both cell wall components as well as glycoproteins in hp*GFT1* lines. The observed relationship between reduced *GFT1* expression, the measured impact on fucosylation in the plant, and the resultant severe developmental defects indicate that GFT1 is likely the sole GDP-Fuc transporter in *Arabidopsis*. Collectively, these data support the notion that GFT1 cannot be substituted by GONST1, GONST2 or GONST3 *in planta.*

Most of the L-fucose in plant cell walls is present in pectin and xyloglucan. The L-fucose content in cell wall extracts of hp*GFT1* lines revealed significant reductions in pectin- and xyloglucan-enriched fractions, while a more detailed analysis of xyloglucan structures indicated reduced levels of L-fucose-containing oligosaccharides. The reduction in cell wall L-fucose, in combination with the developmental abnormalities observed in plant lines with the strongest reduction in *GFT1* expression are reminiscent of the *mur1* mutant^12^,^35^,^36^. *mur1* plants almost completely lack L-fucose due to a loss-of-function mutation in the GMD2 gene, which is required for GDP-Fuc biosynthesis. Similar to our results, *mur1* plants contain significantly reduced levels of L-fucose in pectic fractions^35^ and have decreased L-fucose-containing oligosaccharides and a concomitant increase in non-fucosylated Gal-containing oligosaccharides derived from xyloglucan^12^. It has been previously shown that the *mur1* plant developmental phenotype is specifically associated with an altered structure of RG-II, since other mutations that solely affect fucosylation of xyloglucan or arabinogalactan proteins do not show growth defects under standard growth conditions^6^,^8^,^16^. The hp*GFT1* lines also had a decrease in the amount of L-fucose in *N*-linked glycans. The analysis of *N*-glycans from the *mur1* mutant showed that almost 95% of L-fucose residues were absent^14^. Mutants affected in fucosylation of *N*-glycans such as the hybrid glycan 1 (*hgl1*) or complex glycan 1 (*cgl1*) revealed no major physiological phenotype^37^ besides reduced root growth under salt stress^38^. These observations further support the importance of L-fucose for the correct organization of pectic polysaccharides and their role in plant growth and development. Thus, the severe morphological defects of the hp*GFT1* lines are likely explained by the reduced fucosylation of the pectic polymer RG-II.

The similarity in phenotype between the hp*GFT1* knockdown lines and the *mur1* knockout mutant is likely due to the existence of a second GMD isoform (GMD1) in *Arabidopsis*. While GMD2 is ubiquitously expressed in the aboveground portions of the plant, GMD1 is only expressed in stipules and pollen grains^39^. Therefore, *mur1* mutants are likely to contain residual GDP-Fuc, which enables the plants to survive. This residual functionality in the hp*GFT1* lines also explains the similarities to the *mur1* phenotype. However, it is very likely that the complete lack of *GFT1* expression or function would lead to a complete loss of glycan fucosylation and a lethal phenotype. This is in agreement with our observation that >50% of the *hpGFT1* population (total of 75 independent plant lines) died at early developmental stages. These results strongly suggest that GFT1 may be the only GDP-Fuc transporter in *Arabidopsis*.

It was generally assumed that the structural alterations to RG-II in the *mur1* mutant were mainly due to the substitution of L-fucose by L-galactose^35^; however, it now appears that the altered phenotype is a result of a truncation of chain A from RG-II due to the lack of available GDP-Fuc^9^. It is thus highly likely that the hp*GFT1* lines possess a similar and truncated form of RG-II. Since we observed a significant increase in Ara content, it is possible that the hp*GFT1* plants were compensating RG-II truncations by increasing arabinan side chains on RG-I. Overall, the similarities between the hp*GFT1* lines and the *mur1* mutant support the exclusive role of GFT1 in the transport of GDP-Fuc in *Arabidopsis*. Thus, the impairment of cytosolic-derived GDP-Fuc biosynthesis in plants is akin to the inhibition of GDP-Fuc transport into the Golgi apparatus.

We have identified and characterized a plant Golgi-localized nucleotide sugar transporter that transports GDP-Fuc *in vitro*. This is the first instance where a NST with specificity for GDP-Fuc has been identified in plants. hp*GFT1* mutant plants showed a ∼80% decrease in total cell wall L-fucose content affecting pectins as well as xyloglucans, thus confirming the role of GFT1 in providing GDP-Fuc for cell wall biosynthesis. Fucosylation of glycoproteins was also impaired in hp*GFT1* mutants. Plants with significantly diminished *GFT1* expression were severely dwarfed and did not grow normally, which indicates that GFT1 plays an essential role in plant growth and development.

## Methods

### Substrates

Substrates were obtained from the following sources: UDP-α-D-xylose, UDP-β-L-arabinopyranose and UDP-α-D-galacturonic acid (Carbosource Services, Complex Carbohydrate Research Center, Athens, GA); UDP-α-D-glucuronic acid, UDP-α-D-glucose, UDP-α-D-galactose, UDP-*N*-acetyl-α-D-glucosamine, UDP-*N*-acetyl-α-D-galactosamine, GDP-α-D-mannose, GDP-β-L-fucose, GDP-α-D-glucose, adenosine 3′-phosphate 5′ phosphosulfate, CMP-*N*-acetylneuraminic acid and ADP-α-D-glucose (Sigma-Aldrich, St Louis, MO); and UDP-β-L-arabinofuranose (Peptides International, Louisville, KY). GDP-α-L-Galactose was synthesized enzymatically using the *Arabidopsis* GDP-mannose-3,5-epimerase (GME, At5g28840) essentially as outlined^40^. The GME cDNA was cloned into pET-DEST42 and expressed in *Escherichia coli* BL21 Star (DE3) strain (Thermo Fisher Scientific). The His-tagged GME was then purified from lysates using TALON Metal Affinity Resin (Clontech) and desalted using PD-10 Desalting Columns (GE Healthcare). GDP-Gal was synthesized using 100 μg of purified GME, 10 mM GDP-Man and 50 mM Tris (pH 8.0), and incubated overnight at 25 °C. The resultant GDP-Gal was purified from the remaining GDP-Man and GDP-L-gulose (also biosynthesized by GME) using high-performance liquid chromatography (HPLC) performed using a Dionex Ultimate 3000 with ultraviolet light detection at 262 nm. The reaction was separated on a CarboPac PA20 column (3 × 150 mm; Dionex) at a flow of 0.5 ml min^−1^ with a linear gradient of 50 mM to 1 M ammonium formate over 40 min (ref. 41). Peaks corresponding to GDP-Gal were concentrated using a vacuum concentrator. UDP-β-L-rhamnose was enzymatically synthesized by a two-step reaction from UDP-Glc using the 4,6-dehydratase (RHM-1D) and the 3,5-epimerase 4-reductase (RHM1-ER) domains of *Arabidopsis* UDP-rhamnose synthase (RHM1, At1g78570). The domains were cloned into pET11a (Novagen) and expressed in *E. coli* Rosetta 2 (DE3) strain (EMD Millipore), and purified from lysates using TALON Metal Affinity Resin (Clontech). The initial reaction to produce UDP-D-quinov-4-ulose was synthesized using 1 mM UDP-Glc, 4 μg RHM-1D, 0.5 mM NAD^+^ and 10 mM Tris-HCL (pH 8.5) in a total volume of 0.4 ml, and incubated overnight at 25 °C. The volume of the reaction mixture was adjusted to 2 ml, using a solution containing 1 mM NADPH, 8 μg RHM1-ER and 10 mM Tris-HCl (pH 8.5), and incubated overnight at 25 °C. The resultant UDP-Rha was purified using an ENVI-Carb SPE column (Sigma-Aldrich).

### Sequence analysis

Sequences were retrieved from The *Arabidopsis* Information Resource (TAIR)^42^ or GenBank^43^. Protein sequences were aligned using the ClustalW option with default parameters from Molecular Evolutionary Genetics Analysis (MEGA) version 6.0 (ref. 44). Phylogenetic trees created using the neighbour-joining statistical method and applying the bootstrap method with 1,000 replications and finally visualized using MEGA 6.

### Heterologous expression and transporter assays

Heterologous expression was undertaken using the uracil-auxotrophic *Saccharomyces cerevisiae* (strain INVSc1: *MATa his3D1 leu2 trp1-289 ura3-52 MAT his3D1 leu2 trp1-289 ura3-52*, Thermo Fisher Scientific). Microsomal membranes were isolated from 500-ml cultures grown at 30 °C under constant shaking. The yeast pellet was resuspended in 10 ml resuspension buffer (50 mM potassium phosphate, pH 7.1, 1.4 M sorbitol, 10 mM NaN_3_ and 40 mM 2-mercaptoethanol). To this, 6,000 units Lyticase (Sigma-Aldrich) was added and cells incubated for 1 h at 37 °C. Resultant spheroplasts were collected at 2,500*g* for 5 min, washed once with 0.8 M sorbitol, 10 mM triethanolamine/acetic acid (pH 7.2), 1 mM EDTA and then lysed using acid-washed glass beads (Sigma-Aldrich) in 5 ml 0.8 M sorbitol, 10 mM triethanolamine/acetic acid (pH 7.2) and 1 mM EDTA, supplemented with a protease inhibitor cocktail (Sigma-Aldrich) and 1 mM PMSF. The microsomal fraction was obtained by sequential centrifugation (8,000*g* for 10 min (F1), and 100,000*g* for 75 min (F2)). The F2 fractions were resuspended in reconstitution buffer (10 mM Tricine-KOH (pH 7.5), 50 mM potassium gluconate and 20% glycerol). Reconstitution of yeast microsomal proteins into liposomes was undertaken by the detergent solubilization and rapid removal approach. Approximately 600 mg of acetone washed soybean L-α-phosphatidylcholine (Avanti Polar Lipids) was dissolved in 10 ml chloroform and evaporated under vacuum at 42 °C for 45 min. The resultant film was suspended in reconstitution buffer (10 mM Tricine-KOH (pH 7.5), 50 mM potassium gluconate and 20% glycerol). Microsomal reconstitution was undertaken using around 400 μg microsomal protein in reconstitution buffer, lipid at a ratio of 13 (lipid:protein), 10 mM exchange substrate and 50 mM octyl-β-glucoside. Unincorporated components were removed from reconstituted liposomes using Sephadex G50 (GE Healthcare). Aliquots of 200 μl were incubated with nucleotide sugar substrates at 25 °C for indicated times to assess transporter activities. Kinetic parameters were calculated by non-linear regression using the Prism 6 application (GraphPad Sofware, La Jolla, CA). Polyacrylamide gel electrophoreses was undertaken using 2.5 μg protein of proteo-liposomes separated on a 7–15% SDS–polyacrylamide gel electrophoresis gel. The resolved proteins were transferred onto nitrocellulose (Hybond ECL, GE Healthcare), and immunoblotting was conducted with the anti-V5 antibody using a dilution of 1:10,000 (#R96025, Thermo Fisher Scientific). Detection was accomplished with an anti-mouse IgG-peroxidase secondary antibody (#A4416, Sigma-Aldrich) at a dilution of 1:20,000 in conjunction with the ECL Prime western blotting detection reagent (GE Healthcare) and a ChemiDoc Imaging Systems (Bio-Rad).

### Protein quantification by multiple reaction monitoring

Heterologous expression in yeast was undertaken using the pYES-DEST52 vector, which contains an in-frame V5-tag. Absolute quantification of expressed proteins in yeast proteo-liposomes was undertaken by multiple reaction monitoring (MRM) analysis of a common tryptic peptide (R.SRGPFEGKPIPNPLLGLDSTR.T) to the V5-tag region using a 6460 Triple Quad LC/MS system (Agilent Technologies, CA). A synthetic peptide was used to determine the following parameters for MRM: Dwell (25 msec), Fragmentor (130 V), Collision Energy (11.1 V) and Cell Accelerator Voltage (7 V). Proteo-liposomes (50 μg) containing expressed NSTs were digested with trypsin (5 μg) in 25 mM ammonium carbonate and 50% methanol (v/v), and incubated overnight at 37 °C. Resultant peptides were concentrated and resuspended in water containing 0.1% formic acid before analysis by LC-MS/MS. The system was operated in positive ion mode using the MRM scan type with both MS1 and MS2 set to Unit resolution and the following parameters: gas temperature (350 °C), gas flow (10 l min^−1^), nebulizer (45 p.s.i.), sheath gas temperature (400 °C), sheath gas flow (11 l min^−1^), capillary (5,000 V) and MS1/MS2 heater (100 °C). A total of 10 μg of peptides was loaded onto a Ascentis Express Peptide ES-C18 (5 cm × 2.1 mm, 2.7 μm) column (Sigma-Aldrich) using a 1290 series HPLC (Agilent Technologies) at a flow rate of 0.4 ml min^−1^ as follows: 95% buffer A (99.9% water and 0.1% formic acid) and 5% buffer B (99.9% acetonitrile and 0.1% formic acid) for 0.2 min, followed by an increase to 35% buffer B over 5.5 min, then 90% buffer B in 0.3 min, where it was held for 2 min and back to 5% buffer B over 5 min, giving total runtime of 13 min. The column temperature was maintained at 60 °C. Data were acquired using MassHunter Workstation Software Version B.06.00 Build 6.0.6025.4 SP4 (Agilent Technologies). The synthetic peptide standard was used to create a standard curve by linear regression using a range of abundances (0.5–10 pmol). Resultant data were imported into Skyline (v2.5.0.6157)^45^ and peaks manually inspected for retention time and adjusted accordingly. The abundance of an expressed protein in the sample (10 μg) was calculated by integrating the total signal peak area (total area) from Skyline for two transitions on the 563.560 [M+4H]^4+^ precursor ion, namely, L [*y*7] 761.452 [M+H]^1+^ and G [*y*6] 648.3311 [M+H]^1+^ and calculating total moles in the sample against the standard curve. The expressed NSTs comprise ∼0.5% of total protein in a standard proteo-liposome preparation (Supplementary Table 1). These values were used for the kinetic calculations outlined in Table 1.

### Plant growth and transformation

*Arabidopsis thaliana* (L.) Heynh. Columbia-0 (Col-0) was obtained from the *Arabidopsis* Biological Resource Center (http://abrc.osu.edu/). The T-DNA insertion mutants for *GFT1* (SALK_094857; SAIL_400_E04) were obtained from the SIGnAL Salk collection^46^ and Syngenta *Arabidopsis* Insertion Library (SAIL) collection^47^. A hairpin construct specifically targeting *GFT1* expression was assembled using the pHANNIBAL/pART27 system^48^ and designated hp*GFT1*. A 506-bp fragment was amplified from genomic DNA using the following primer pairs: hpGFT1s_fwd (5′-acggaattccgAAGTCGTCGCCTTCCTCTAATT-3′), hpGFT1s_rev (5′-ggggtaccccTCGAAGGTAAAGGCTGACTACGA-3′) and hpGFT1as_fwd (5′-agctctagagcAAGTCGTCGCCTTCCTCTAATT-3′), hpGFT1as_rev (5′-ccatcgatggTCGAAGGTAAAGGCTGACTACGA-3′), and cloned into the pHANNIBAL vector in sense and antisense orientation at the EcoRI to Asp718 and XbaI to ClaI restriction sites. The expression cassette was subsequently excised from the pHANNIBAL vector using NotI and sub-cloned into the corresponding Not1 site of the plant transformation vector pART27. For kanamycin selection, seeds were germinated on half-concentrated MS medium^49^ supplemented with 1% (w/v) sucrose, 75 μg ml^−1^ kanamycin, 100 μg ml^−1^ Cefotaxime (Sigma-Aldrich) and solidified with 0.7% (w/v) agar under a 16-h photoperiod (120 μmol m^−2^ s^−1^, 22 °C). Plants were transferred to soil (PRO-MIX, Premier Horticulture Inc.) and further grown in an *Arabidopsis* growth chamber (Percival-Scientific) under short-day light conditions (10 h of fluorescent light (120 μmol m^−2^ s^−1^) at 22 °C and 60% relative humidity (RH)/14 h of dark at 22 °C and 60% RH). After 4 weeks plants were transferred to long-day conditions (16 h of fluorescent light (120 μmol m^−2^ s^−1^) at 22 °C and 60% RH/8 h of dark at 22 °C and 60% RH).

### Cloning procedures

Coding sequences for *Arabidopsis GONST3* and *GFT1* without the native stop codon were PCR amplified from genomic DNA and *GONST1* was PCR amplified from leaf cDNA using the following primer pairs: GONST3-fwd (5′-CACCATGTCGACGAATGATGAGGAAAATGG-3′), GONST3-rev (5′-TAGTTTCTCTTCTGATTTCAGAGTTTCCTT-3′); GFT1-fwd (5′- CACCATGTCGTCCTCTCGATTCGATTCAA-3′), GFT1-rev (5′-TACAACAGAAGCTAGTTTCCCCGG-3′); and GONST1-fwd (5′-CACCATGAAATTGTACGAACACGATGGAGTTGA-3′), GONST1-rev (5′-GGACTTCTCCCTCATTTTGGCTCTAGCA-3′). *GONST2* was synthesized according the current gene model (TAIR10; GenScript, Piscataway, NJ) and amplified using the following primer pair GONST2-fwd (5′-CACCATGTCTGCCGTGAAACTGGAAGC-3′) and GONST2-rev (5′-TGACATTTTAGCTCTGGCAAAGACCACT-3′). To generate the respective entry clones, the resultant PCR products were introduced into the pENTR/SD/D-TOPO cloning vector (Life Technologies) according to the instructions of the manufacturer. C-terminal YFP fusions were created by introduction of the constructs into the 35S promoter carrying pEarleyGate101 plant transformation vector^50^, using the LR Clonase II reaction (Life Technologies) following the manufacturer's protocol. To obtain yeast expression clones, the constructs were subsequently cloned into the yeast expression vector pYES-DEST52 (Life Technologies) using LR Clonase II (Life Technologies).

### Sub-cellular localization and microscopy

For transient protein expression in *N. benthamiana* (tobacco) leaves, *Agrobacterium tumefaciens* strain GV3101 pmp90 carrying the C-terminal YFP fusion constructs and the aMan-mCherry Golgi marker (G-rk) or the mCherry ER marker (ER-rk)^51^ were grown overnight in Luria-Bertani (LB) media. After a centrifugation step (10 min, 4,000*g*, 15 °C), the supernatant was discarded and the cell pellets were resuspended in infiltration buffer containing 10 mM MES, 10 mM MgCl_2_ and 100 μM acetosyringone to an OD_600_=0.01–0.15. Subsequently, leaves of 4-week-old *N. benthamina* plants (grown at 25/24 °C day/night temperature, 60% humidity and 16-h light/8-h dark cycles) were co-infiltrated with the *Agrobacterium* mixtures in infiltration buffer using a 1-ml syringe. After infiltration, plants were moved back to the growth room and protein expression was monitored 2 days post infiltration. Visualization by confocal laser scanning microscopy was performed using excitation wavelengths of 514 nm (YFP) and 580 nm (mCherry). Emissions were collected at 520–560 nm (YFP) and 600–630 nm (mCherry). The pinhole diameter was set at 1 Airy unit and a × 63 1.4 numerical aperture oil immersion objective was used. Raw images were processed using ImageJ^52^. Background signals were subtracted using negative control images taken from the same experimental material. The Co-localization threshold tool in ImageJ was used to calculate the Manders' tM1 and tM2 overlap coefficients on regions of interest to quantify co-localization between the fluorescently labelled proteins.

### Categorization of hp*GFT1* plants

The 4-week-old T1 hp*GFT1* plants were divided into four cohorts based on the rosette size when compared with the vector control lines. The hp*GFT1* plants with rosettes >50% of control lines were assigned as cohort #1, plants with rosettes ∼50% were assigned as cohort #2, plants with rosettes ∼25% were assigned as cohort #3 and plants with rosettes <25% were assigned as cohort #4. Due to limitations in plant tissue from cohorts #3 and #4, plant material for all downstream analyses was derived from 5 to10 pooled individuals for all lines.

### Cell wall monosaccharide composition

Plant material frozen in liquid nitrogen was ground to a fine powder before boiling in 96% ethanol for 30 min. After a centrifugation step of 5 min at 20,000*g*, the supernatant was discarded. The resultant pellet was washed with 70% ethanol until chlorophyll was removed. Last, the pellet was washed with 100% acetone and dried in a vacuum concentrator. Samples were hydrolysed in 2 N trifluoroacetic acid (TFA) for 1 h at 120 °C. High-performance anion exchange chromatography with pulsed amperometric detection was performed on an ICS 3000 (Dionex Corporation, Sunnyvale, CA) using a CarboPac PA20 (3 × 150 mm, Dionex Corporation, Sunnyvale, CA) anion exchange column at a flow rate of 0.4 ml min^−1^. Before sample injection, the column was equilibrated with 10 mM NaOH for 10 min. The elution program involved two isocratic elution steps with 10 mM NaOH from 0 to 15 min to separate the neutral sugars followed by a ramp step to 450 mM NaOH from 15.1 to 35 min, which allowed separation of uronic acids and washing of the column. Monosaccharide standards comprised L-Fuc, L-Rha, L-Ara, CGal, D-Glc, D-Xyl, _D_-GalA and D-GlcA. A run of a standard mixture containing the eight monosaccharides was performed with each sample set to enable sample quantitation by linear regression.

### Mutant identification by PCR

To identify homozygous T-DNA insertion lines, PCR was performed using the following primers: SAIL_400_E04 (5′-TTCCATTAATGGTGAGTTGACC-3′) (fwd) and (5′-CTGCTGATAACCAACACCTCC-3′) (rev), and SALK_094857 with (5′-AGCGTTGGTTCAGCACATATC-3′) (fwd) and (5′-AGGAAACCAAACACACACGAC-3′) (rev). For the PCR verifying the T-DNA, the SAIL_LB (5′-GCCTTTTCAGAAATGGATAAATAGCCTTGCTTCC-3′) and the SALK_LBb1.3 (5′-ATTTTGCCGATTTCGGAAC-3′) were used.

### Quantitative PCR

RNA was extracted from plant tissue using the RNeasy RNA Plant kit (Qiagen) and a total of 0.5–1 μg was reverse-transcribed using SuperScriptII reverse transcriptase (Thermo Fisher Scientific) and d(T)_15_ oligomers (Life Technologies). To determine *GFT1* and *GONST3* expression in hp*GFT1* lines, resultant cDNA was subsequently used as template in a reverse transcription qPCR reaction containing 2 × SYBR Select Master Mix (Applied Biosystems) and gene-specific primers. *GFT1* was amplified with (5′-GCGTCAACTCCTCATCTTCC-3′) (fwd) and (5′-TAAGCATGCCACTCCTGTTG-3′) (rev), and *GONST3* with (5′-GGAAACGCTTTTCTTGCATC-3′) (fwd) and (5′-ACGCAAGAGCCCAGCTATAA-3′) (rev). As a reference, the *Arabidopsis* ubiquitin-10 gene (At4g05320) was amplified using the primers (5′-GGCCTTGTATAATCCCTGATGAATAAG-3′) (fwd) and (5′-AAAGAGATAACAGGAACGGAAACATAGT-3′) (rev), and the Arabidopsis *PP2A* gene (At1g13320) was amplified using the primers (5′-TAACGTGGCCAAAATGATGC-3′) (fwd) and (5′-GTTCTCCACAACCGCTTGGT-3′) (rev). The PCR reactions were performed using the StepOnePlus Real-Time PCR (Applied Biosystems) system and the following thermal profile was used for all PCR reactions: 50 °C for 2 min, 95 °C for 10 min, 40 cycles of 95 °C for 15 s and 60 °C for 1 min. Amplicon dissociation curves (melting curves) were recorded after cycle 40 by heating from 60 to 95 °C with a ramp speed of 1.9 °C min^−i^. Data were analysed using the SDS 2.2.1 software (Applied Biosystems). Relative expression levels were determined using the geNorm algorithm^53^, which uses the geometric mean of the housekeeping genes (UBI10 and PP2A).

### Protein extraction and immunoblotting

Protein was extracted from plant material by grinding in extraction buffer (10 mM Tris (pH 8), 150 mM NaCl, 2% Triton, 1 mM PMSF, protease inhibitor and 10 mM CaCl_2_) and incubated for 1 h at 4 °C under constant shaking. The homogenate was centrifuged for 30 min at 20,000*g* at 4 °C to remove cell debris. Proteins were precipitated with 20% trichloroacetic acid after incubation on ice and centrifuged at 20,000*g* at 4 °C. After removal of the supernatant, samples were washed twice with ice-cold acetone and dried using a vacuum concentrator. For immunoblotting, proteins were suspended in SDS sample buffer and separated by SDS–polyacrylamide gel electrophoresis (7–15%) and transferred onto nitrocellulose (Hybond ECL, GE Healthcare). *N*-glycan epitopes were detected using antibodies raised against ß-(1,2)-xylose (#AS07 267) and α-(1,3)-fucose (#AS07 268; Agrisera, Sweden) using dilutions1:10,000 and 1:5,000, respectively. Detection was accomplished with an anti-rabbit IgG-peroxidase secondary antibody (#A9169, Sigma-Aldrich) at a dilution of 1:20,000 in conjunction with the ECL Prime western blotting detection reagent (GE Healthcare) and a ChemiDoc Imaging System (Bio-Rad).

### Oligosaccharide mass profiling

Plant tissue was frozen in liquid nitrogen and homogenized using a mixer mill (Retsch), resuspended in 96% ethanol, and incubated at 100 °C for 30 min. The pellet was then collected by centrifugation (20,000*g* for 5 min) and subjected to three wash steps: two washes with 70% ethanol, followed by a wash with 100% acetone. The remaining pellet was dried in a vacuum concentrator. Samples were digested with a xyloglucan-specific endoglucanase from *Aspergillus aculeatus*^36^ in 50 mM ammonium formate (pH 4.5), for 16 h at 37 °C. Following digestion, the solubilized xyloglucan oligosaccharides were collected by centrifugation (1,000*g* for 5 min) and desalted using the BioRex MSZ 501 cation exchange resin beads (Bio-Rad). A total of 2 μl of the desalted sample was spotted onto a MALDI plate with 2,5-dihydroxybenzoic acid and analysed using an AXIMA (Shimadzu Corporation) matrix-assisted laser desorption/ionization time-of-flight set in linear positive mode with an acceleration voltage of 20,000 V.

### Extraction of nucleotide sugars

Frozen plant material (∼40 mg) was homogenized using a mixer mill (Retsch) and 600 μl of ice-cold chloroform/methanol solution (30% chloroform/70% methanol) added to each sample. The homogenate was incubated at −20 °C for 2 h, then 400 μl ice-cold water was added and samples were mixed and centrifuged at 20,000*g* for 5 min at 4 °C. The upper phase was transferred to a 15-ml tube. This step was repeated twice and the combined upper phases were lyophilized overnight. The dried extracts were dissolved in 1 ml 10 mM ammonium bicarbonate before purification using ENVI-Carb SPE columns (Sigma-Aldrich). Eluates were lyophilized overnight, resuspended in ice-cold water and analysed by LC-MS/MS as described below.

### Analysis of nucleotide sugars by tandem mass spectrometry

Tandem mass spectrometry (LC-MS/MS) was performed at a flow rate of 50 μl min^−1^ using a Hypercarb column (150 mm × 1 mm, 5 μm), as the stationary phase with an 1100 series HPLC system (Agilent Technologies) and a 4000 QTRAP LC-MS/MS system (Sciex, CA) equipped with a TurboIonSpray ion source. The system was run in Micro mode with a mix rate of 400 μl min^−1^ and the column compartment was set to 50 °C with samples kept at 4 °C. Initial conditions were 95% buffer A (LC-MS grade water with 0.3% formic acid, pH 9.0 with ammonia) and 5% buffer B (100% acetonitrile) for 1 min followed by a gradient to 75% (A) in 20 min, then 50% (A) in 5 min before returning to 95% (A) in 5 min. Specific compound-dependent MS parameters for each nucleotide sugar were determined previously^20^. The 4000 QTRAP was operated in negative ion mode using the MRM scan type. A declustering potential of −40, entrance potential of −10, collision cell exit potential was −15. The ion spray voltage was set at −4,200 V, source temperature (TEM) at 425 °C, collision gas (CAD) was set to high and source gas 1 (GS1) and 2 (GS2) were both set to 20. A time of 100 ms was applied for each transition resulting in a duty cycle of 1.0501, s with both Q1 and Q3 resolutions set to Unit. All data were acquired using Analyst 1.5.1 Build 5218 (Sciex, CA) operating in MRM mode. Nucleotide sugars were quantified using MultiQuant 2.1 (build 2.1.1296.02.1) software (Sciex, CA) by integrating the signal peak areas.

### Data availability

The nucleotide sequences for cloned *Arabidopsis* GONST family members have been deposited in the GenBank with accession codes KR265320 (At2g13650, *GONST1*), KR265321 (At1g07290, *GONST2*) and KR265322 (At5g19980, *GFT1*). All other data supporting the findings of this study are available within the article and its Supplementary Information files or from the corresponding author on request.

## Additional information

**How to cite this article:** Rautengarten, C. *et al.* The *Arabidopsis* Golgi-localized GDP-L-fucose transporter is required for plant development. *Nat. Commun.* 7:12119 doi: 10.1038/ncomms12119 (2016).

## Supplementary Material

Supplementary Information Supplementary Figures 1 - 3 and Supplementary Tables 1 - 4

## Figures and Tables

**Table 1:** Kinetic parameters of GDP-Fuc and GDP-Man transport into proteo-liposomes.

**Parameter**	**GDP-Fuc**		**GDP-Man**
	**GFT1**	**GONST1**	**GONST1**
*K*_m_ (μM)	7 (1)	76 (5)	17 (3)
*V*_max_ [nM s^−1^]	54 (1)	14 (0)	24 (1)
*k*_cat_ (s^−1^)	4.0	0.7	1.2

For each transporter (GFT1 or GONST1), data points with varying substrate concentrations (0.5–400 μM) were acquired. Values are mean (s.e.m.) of *n*=4.
