# Supplementary material for: The Arabidopsis Golgi-localized GDP-L-fucose transporter is required for plant development
Source: Nat Commun. 2016 Jul 6;7:12119. doi: 10.1038/ncomms12119 (PMC4935801; doi:10.1038/ncomms12119)
Supplement: Supplementary Information — Supplementary Figures 1 - 3 and Supplementary Tables 1 - 4 [file ncomms12119-s1.pdf]

The Arabidopsis Golgi-localized GDP-L-fucose transporter is required for plant development

Carsten Rautengarten, Berit Ebert, Lifeng Liu, Solomon Stonebloom, Andreia M. Smith-Moritz, Markus Pauly, Ariel Orellana, Henrik Vibe Scheller, Joshua L. Heazlewood

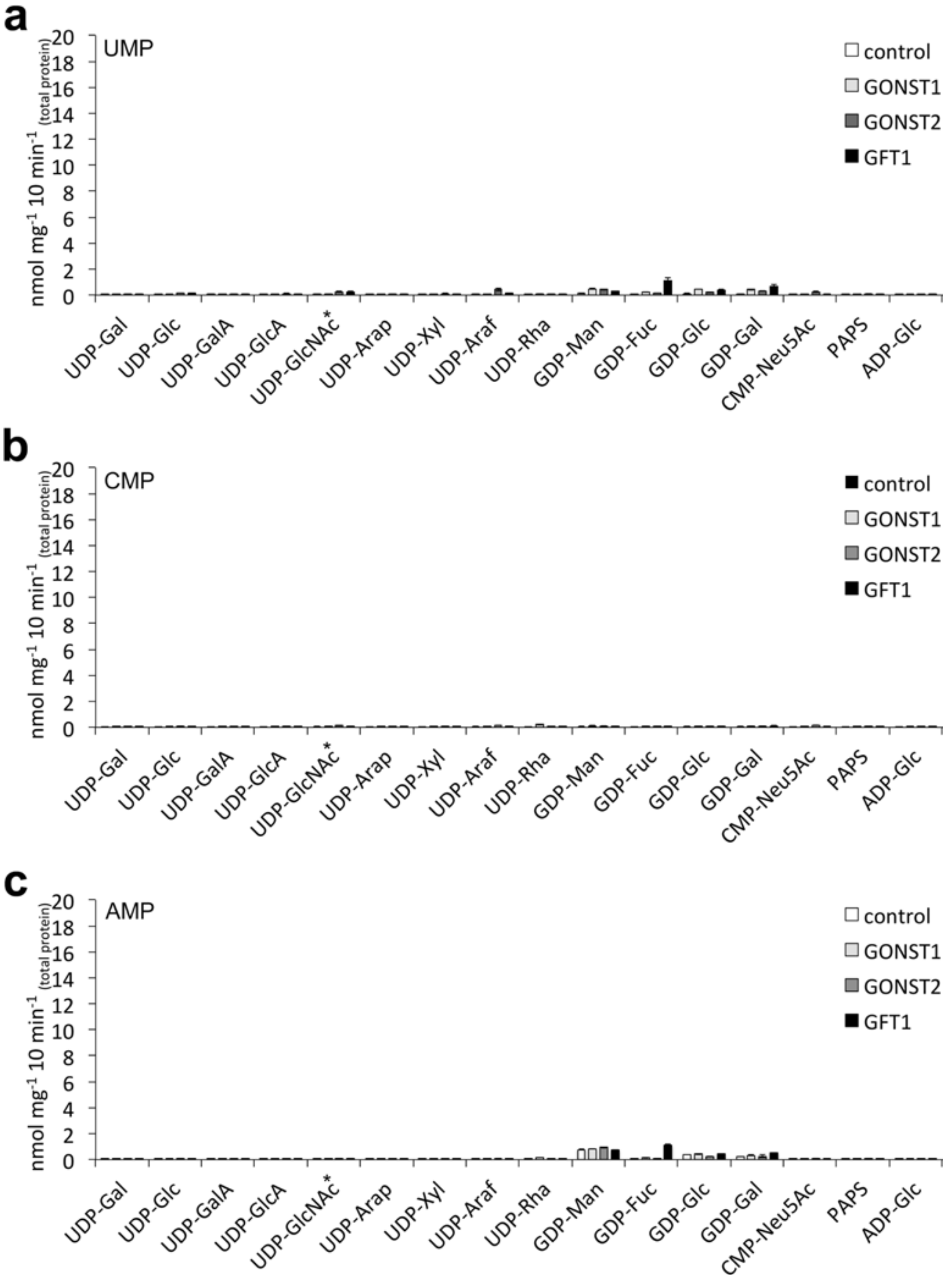

Supplementary Figure 1. Exchange substrate specificities of GFT1, GONST1 and GONST2.

Proteo-liposomes derived from yeast transformed with the empty vector (control) or yeast expressing GONST1, GONST2, or GFT1 were pre-loaded with **(a)** 10 mM UMP, **(b)** 10 mM CMP, or **(c)** 10 mM AMP and incubated with 16 nucleotide sugar substrates. Only minor transport was observed when compared to proteoliposomes pre-loaded with 10 mM GMP (Figure 3).

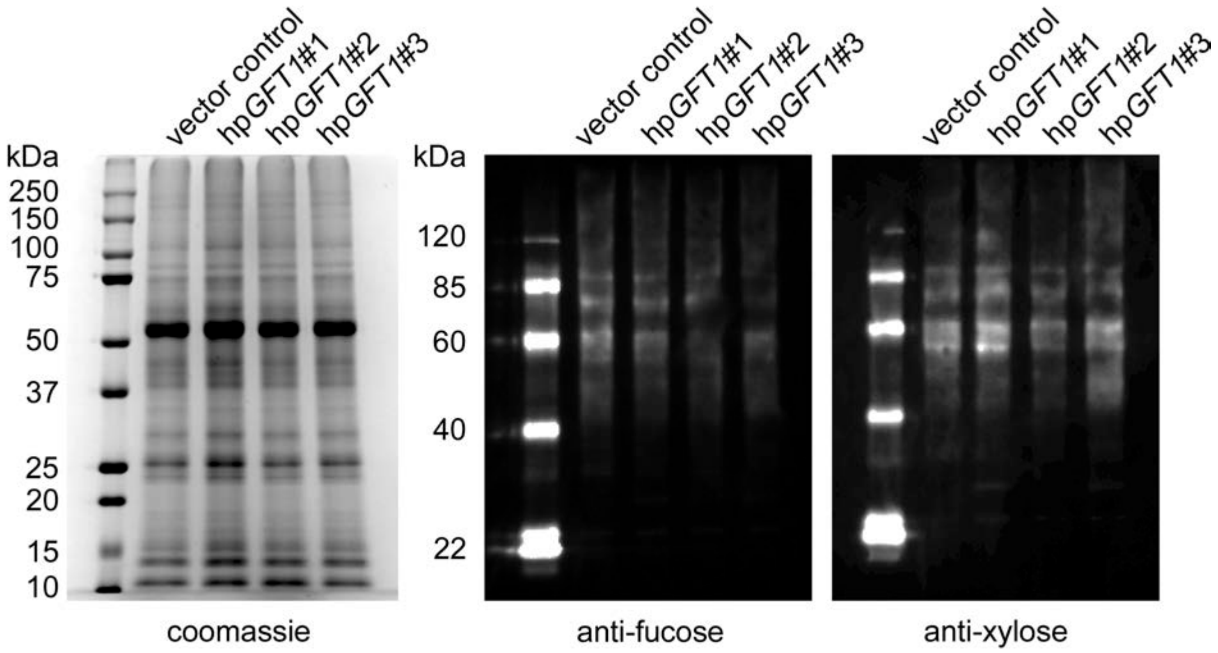

Supplementary Figure 2. lmmunoblot analysis and Coomassie stained gel of total protein extracted from hp*GFT1* cohorts probed with antibodies against *N*-glycan xylosyl and fucosyl epitopes.

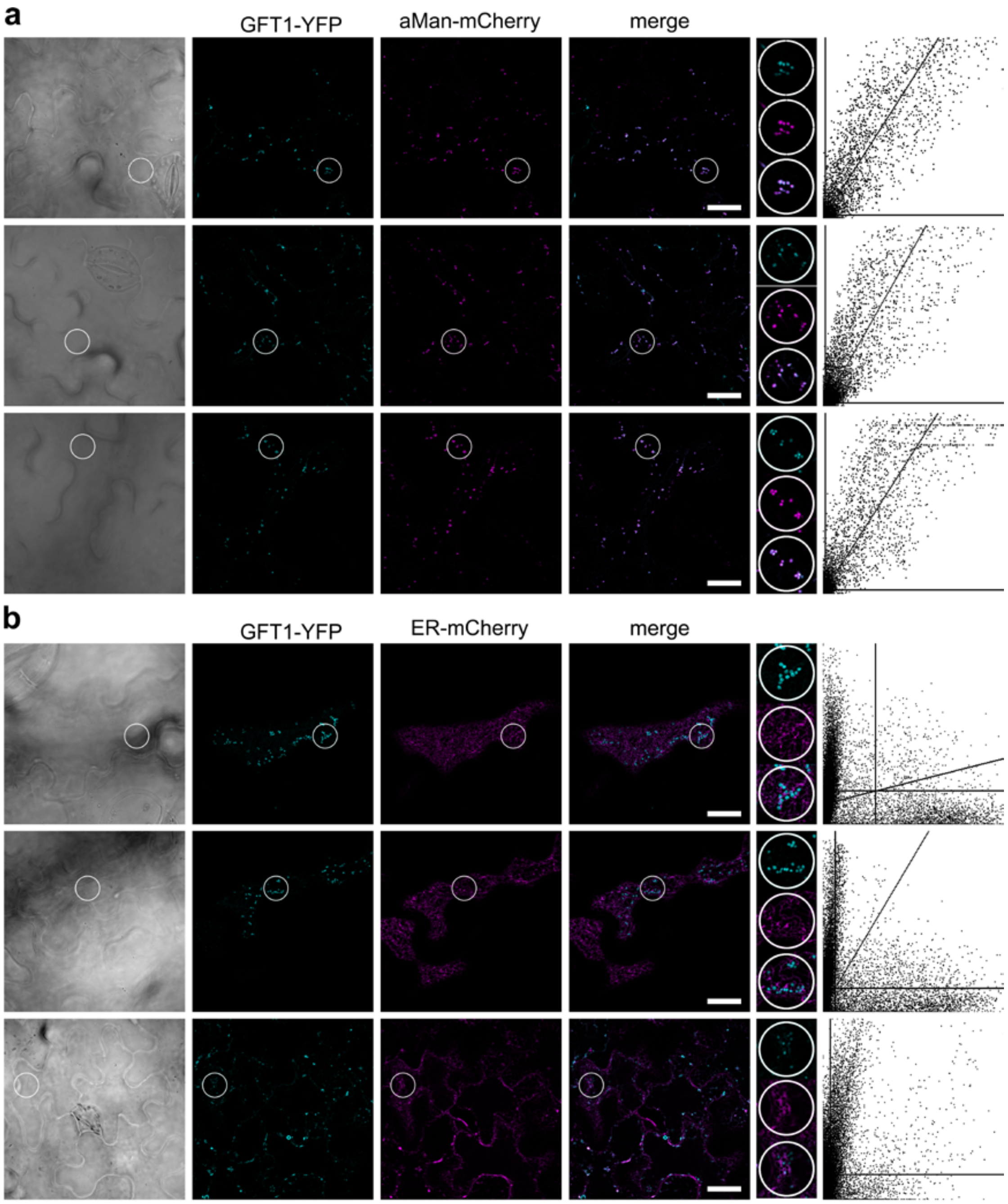

c

	aMan-mCherry tM1	aMan-mCherry tM2	ER-mCherry tM1	ER-mCherry tM2
Image 1	83	84	26	6
Image 2	89	89	36	22
Image 3	85	83	53	51
mean	86	85	38	26
s.e.m.	2	2	8	13

Supplementary Figure 3. Confirmed localization of GFT1.

Transient sub-cellular co-localization of the GFT1-YFP fusion protein with the **(a)** G-rk (aMan-mCherry) Golgi marker or the **(b)** ER-rk (ER-mCherry) ER marker in *N. benthamiana* leaves. Regions of interest (cicles) were used to assess the overlap of pixels in each channel to generate scatter plots. The x-axis represents channel 1 (YFP) and the y-axis channel 2 (mCherry). Three independently transformed cells are shown for each experiment. Scale bars = 25 µm. **(c)** Quantification of fluorescent signal overlap in the region of interest (circles) between the GFT1-YFP signal and the organelle marker signals using the Colocalization Threshold tool in lmageJ to calculate the Manders' tM1 and tM2 overlap coefficients.

Supplementary Table 1. Calculations for amount of expressed protein in proteo-liposomes used for transport assays.

NST	Molecular Mass (Da)	fmol (in 10 µg)	ng (in 10 µg)	Total protein (%)
GFT1	42,259	764.7 ± 36.0	32.3 ± 1.5	0.32 ± 0.02
GONST1	41,713	1123.9 ± 49.3	46.9 ± 2.1	0.47 ± 0.02

The molecular mass is the estimated monoisotopic mass including the V5-tag and 6-His tag using the Compute pI/Mw tool at ExPASy (http://web.expasy.org/). The amount (fmol) in the sample was estimated using LC-MS/MS (MRM) quantitation of a shared C-terminal peptide (SRGPFEGKPIPNPLLGLDSTR). Results are mean (*n*=2) ± s.e.m. from the proteo-liposome preparations used for the transporter assay.

Supplementary Table 2. Nucleotide sugar content of hp*GFT1* plants and empty vector control plants.

compound	vector control	hpGFT1#1	hpGFT1#2	hpGFT1#3	hpGFT1#4
UDP-α-D-Glc	61.0 ± 1.5	59.8 ± 0.2	59.8 ± 1.5	57.7 ± 0.8	58.6 ± 2.3
UDP-α-D-Gal	15.1 ± 0.8	15.0 ± 0.2	15.0 ± 0.7	15.9 ± 0.7	14.8 ± 1.2
UDP-β-L-Rha	3.3 ± 0.2	3.1 ± 0.1	3.1 ± 0.3	3.4 ± 0.2	3.3 ± 0.2
UDP-α-D-GlcA	4.7 ± 0.7	5.6 ± 0.2	5.6 ± 0.5	5.2 ± 0.4	5.0 ± 0.6
UDP-α-D-GalA	3.1 ± 0.2	3.2 ± 0.1	3.2 ± 0.1	2.8 ± 0.0	3.2 ± 0.0
UDP-α-D-Xyl	3.4 ± 0.2	3.9 ± 0.4	3.9 ± 0.3	4.1 ± 0.2	4.0 ± 0.1
UDP-β-L-Ara*p*	2.2 ± 0.2	2.8 ± 0.3	2.8 ± 0.0	3.5 ± 0.1	3.3 ± 0.1
UDP-β-L-Ara*f*	0.7 ± 0.0	0.7 ± 0.2	0.7 ± 0.0	1.0 ± 0.2	1.0 ± 0.1
UDP-α-D-GlcNAc	3.5 ± 0.3	3.2 ± 0.3	3.2 ± 0.4	3.4 ± 0.4	4.0 ± 0.5
GDP-β-L-Fuc	0.3 ± 0.1	0.2 ± 0.0	0.2 ± 0.0	0.3 ± 0.0	0.3 ± 0.0
GDP-α-D-Glc	trace	trace	trace	trace	trace
GDP-α-L-Gal	trace	trace	trace	trace	trace
GDP-α-D-Man	2.8 ± 0.5	2.5 ± 0.2	2.5 ± 0.3	2.6 ± 0.1	2.5 ± 0.3

Values are given in mol% and represent the mean ± s.d. of each hp*GFT1* cohort analyzed in triplicate. No significant differences were observed.

Supplementary Table 3. Monosaccharide composition of sequentially extracted cell wall material from control and hp*GFT1* lines.

**Fraction**	**Fuc** Avg mol%	**Rha** Avg mol%	**Ara** Avg mol%	**Gal** Avg mol%	**Xyl** Avg mol%	**GalA** Avg mol%	**GlcA** Avg mol%
**CDTA**							
control	0.67 (0.11)	5.19 (0.88)	12.53 (1.83)	9.34 (0.08)	4.11 (0.67)	67.70 (3.71)	0.46 (0.47)
hp*GFT1*#1	0.35	4.61	9.99	9.43	2.83	72.65	0.14
hp*GFT1*#2	0.33	5.56	12.07	8.61	3.86	69.52	0.07
hp*GFT1*#3	0.31	5.33	11.76	8.36	4.03	70.11	0.10
hp*GFT1*#4	0.16	4.95	11.92	8.62	3.38	70.83	0.15
average	0.29 (0.09)*	5.11 (0.42)	11.43 (0.97)	8.75 (0.47)	3.52 (0.54)	70.78 (1.36)	0.12 (0.04)
p-value	0.001	0.87	0.29	0.09	0.19	0.14	0.18
**Na_2CO_3**							
control	0.98 (0.12)	9.75 (0.78)	20.54 (3.73)	14.43 (1.58)	6.39 (1.33)	46.56 (7.13)	1.32 (0.27)
hp*GFT1*#1	0.42	9.32	16.77	13.60	5.08	53.55	1.24
hp*GFT1*#2	0.34	10.4	21.32	13.08	5.78	48.15	0.93
hp*GFT1*#3	0.31	9.53	20.94	13.05	6.07	49.35	0.75
hp*GFT1*#4	0.16	11.29	27.05	13.79	6.01	40.92	0.77
average	0.31 (0.1)*	10.13 (0.9)	21.51 (4.22)	13.37 (0.37)	5.73 (0.45)	47.99 (5.25)	0.92 (0.22)
p-value	0.00006	0.52	0.73	0.21	0.35	0.74	0.05
**1N KOH**							
control	2.01 (0.26)	1.32 (0.32)	12.13 (3.84)	11.55 (1.05)	35.64 (4.32)	27.8 (4.05)	9.51 (7.98)
hp*GFT1*#1	1.01	1.25	8.08	11.10	29.40	32.26	16.90
hp*GFT1*#2	0.70	2.15	10.98	12.37	35.92	27.86	10.01
hp*GFT1*#3	0.76	2.04	11.55	12.79	38.95	24.13	9.77
hp*GFT1*#4	0.20	1.54	10.71	13.01	47.19	19.40	7.95
average	0.66 (0.33)*	1.74 (0.42)	10.33 (1.54)	12.31 (0.85)	37.86 (7.38)	25.91 (5.46)	11.15 (3.93)
p-value	0.0007	0.15	0.38	0.27	0.62	0.59	0.70
**4N KOH**							
control	4.89 (1.23)	1.32 (1.18)	4.52 (1.55)	15.2 (0.57)	54.62 (8.43)	17.65 (6.64)	1.76 (0.52)
hp*GFT1*#1	2.33	0.58	4.21	15.60	60.93	15.43	0.93
hp*GFT1*#2	1.27	1.49	7.38	17.12	51.26	20.47	1.01
hp*GFT1*#3	1.32	1.02	6.26	17.67	54.86	15.61	3.27
hp*GFT1*#4	0.00	0.70	5.40	16.81	59.96	16.19	0.94
average	1.22 (0.95)*	0.94 (0.4)	5.81 (1.34)	16.79 (0.87)*	56.75 (4.52)	16.92 (2.38)	1.53 (1.15)
p-value	0.002	0.53	0.23	0.03	0.64	0.83	0.73

Values are shown as mole percent (mol%) of evaluated sugars. The control values are mean (s.d.) of five biological replicates (*n*=5). The data for hp*GFT1* lines are mean values of each cohort analyzed in triplicate. The average is the mean (s.d.) of the data from the four (*n*=4) hpGFT1 lines. The *p*-values between the control and average were calculated using a Student's t-test. The significant differences ($p<0.05$) are marked (*). The Glc values were omitted as a de-starching step was not included in the extraction.

Supplementary Table 4. Relative abundance of xyloglucan oligosaccharides from hp*GFT1* wall material determined by oligosaccharide mass profiling (OLIMP).

Oligosaccharide	vector control	hp*GFT1*#1	hp*GFT1*#2	hp*GFT1*#3	hp*GFT1*#4
XXXG	29.9 (0.9)	28.2 (2.1)	28.2 (2.7)	27.1 (3.2)	26.7 (1.8)
XXLG/XLXG	8.0 (0.4)	22.1 (1.2)*	29.5 (1.2)*	35.8 (0.7)*	38.1 (0.5)*
XX$\underline{L}$G	1.3 (0.4)	0.9 (0.0)	0.8 (0.1)	0.7 (0.2)	0.6 (0.2)
XXFG	10.0 (0.9)	5.1 (0.6)*	2.7 (0.3)*	1.2 (0.1)*	0.5 (0.1)*
XLLG/XXJG/XXFG+	2.7 (0.8)	14.1 (1.6)*	21.0 (1.8)*	27.2 (3.1)*	29.5 (2.0)*
XX$\underline{F}$G	11.5 (2.1)	7.1 (0.8)*	4.1 (0.5)*	1.4 (0.3)*	0.7 (0.1)*
XL$\underline{L}$G/XX$\underline{J}$G/XX$\underline{F}$G+	1.9 (0.9)	2.0 (0.6)	2.2 (0.2)	2.2 (0.2)	2.2 (0.5)
XLFG	14.6 (2.3)	7.7 (0.8)*	4.3 (0.6)*	1.7 (0.5)*	0.7 (0.1)*
XLJG/XLFG+	trace	trace	trace	trace	trace
XL$\underline{F}$G	20.2 (1.4)	12.9 (1.1)*	7.3 (0.5)*	2.7 (0.4)*	0.9 (0.1)*
XL$\underline{J}$G/XL$\underline{F}$G+	trace	trace	trace	trace	trace
fucosylated XyG	56.2 (2.4)	32.7 (1.4)*	18.3 (0.4)*	7.1 (0.6)*	2.9 (0.1)*

All xyloglucan oligosaccharide masses are $[M + Na^{+}]$, except those labelled (+) which indicates $[M + K^{+}]$. One letter code nomenclature of oligosaccharides according to (Fry et al. 1993). Suggested likely oligosaccharide structure is based on the m/z of the ion. "Fucosylated XyG" represents the sum of the abundance of XXFG, XXFG, XLFG, and XLFG. Data are mean ± (s.e.m.) of each hp*GFT1* cohort analyzed in triplicate, Student's *t*-test $p<0.05$ (*).
